# Enhancing accreditation outcomes for medical laboratories on the Strengthening Laboratory Management Toward Accreditation programme in Kenya via a rapid results initiative

**DOI:** 10.4102/ajlm.v11i1.1614

**Published:** 2022-05-31

**Authors:** Ernest P. Makokha, Raphael O. Ondondo, Daniel K. Kimani, Thomas Gachuki, Frank Basiye, Mercy Njeru, Muthoni Junghae, Marie Downer, Mamo Umuro, Margaret Mburu, Jane Mwangi

**Affiliations:** 1 Laboratory Services Branch, Division of Global HIV & TB, United States Centers for Disease Control and Prevention, Nairobi, Kenya; 2 National HIV Reference Laboratory, Division of Public Health Laboratories, Ministry of Health, Nairobi, Kenya

**Keywords:** rapid results initiative, accreditation, quality systems essentials, SLIPTA, SLMTA, e-SLIPTA checklist

## Abstract

**Background:**

Since 2010, Kenya has used SLIPTA to prepare and improve quality management systems in medical laboratories to achieve ISO 15189 accreditation. However, less than 10% of enrolled laboratories had done so in the initial seven years of SLMTA implementation.

**Objective:**

We described Kenya’s experience in accelerating medical laboratories on SLMTA to attain ISO 15189 accreditation.

**Methods:**

From March 2017 to July 2017, an aggressive top-down approach through high-level management stakeholder engagement for buy-in, needs-based expedited SLIPTA mentorship and on-site support as a rapid results initiative (RRI) was implemented in 39 laboratories whose quality improvement process had stagnated for 2–7 years. In July 2017, SLIPTA baseline and exit audit average scores on quality essential elements were compared to assess performance.

**Results:**

After RRI, laboratories achieving greater than a 2-star SLMTA rating increased significantly from 15 (38%) at baseline to 33 (85%) (*p* < 0.001). Overall, 34/39 (87%) laboratories received ISO 15189 accreditation within two years of RRI, leading to a 330% increase in the number of accredited laboratories in Kenya. The most improved of the 12 quality system essentials were Equipment Management (mean increase 95% CI: 5.31 ± 1.89) and Facilities and Biosafety (mean increase [95% CI: 4.05 ± 1.78]) (both: *p* < 0.0001). Information Management and Corrective Action Management remained the most challenging to improve, despite RRI interventions.

**Conclusion:**

High-level advocacy and targeted mentorship through RRI dramatically improved laboratory accreditation in Kenya. Similar approaches of strengthening SLIPTA implementation could improve SLMTA outcomes in other countries with similar challenges.

## Introduction

In the recent past, low- and middle-income countries have witnessed improved access to quality-assured laboratory diagnosis, leading to better patient outcomes.^1^ One of the key drivers for this improvement was the implementation of the twin Strengthening Laboratory Management Toward Accreditation (SLMTA) task-based training programme and the World Health Organization’s (WHO) Stepwise Laboratory Quality Improvement Process Towards Accreditation (SLIPTA), a stepwise accreditation preparedness programme.^2,3^ Used in combination, the SLMTA programme, the SLIPTA checklist and related processes enabled laboratories in low- and middle-income countries to implement quality systems improvements and prepare them for accreditation: a formal recognition of the implementation of laboratory quality systems and services that adhere to international standards.^4^ International Standards Organization (ISO) 15189 is the standard of choice for accreditation of clinical laboratories, as it promotes the delivery of reliable results for patient safety and care, as well as reliable data for public health interventions.^5^

Kenya adopted the SLMTA programme in 2010 to guide laboratory quality improvements prior to accreditation by the national accreditation body, Kenya National Accreditation Service. The SLMTA programme was designed to span between 12 and 18 months, to include three workshops spaced between quality management systems (QMS) implementation, and to have participants implement improvement projects after each workshop, supported by regular supervisory mentorship visits or on-site mentoring. Audits are conducted at the beginning and end of the training using the SLIPTA checklist to assess strengths, weaknesses, and progress made.^6^ Based on the assessments, laboratories are scored from 0 to 5 stars. Laboratories scoring 3 stars and above are eligible to apply to accreditation institutions to commence the formal accreditation assessment process. By February 2020, after ten years of SLMTA implementation, 52 countries, 23 in Africa, had implemented the SLMTA programme in more than 1000 laboratories with 220 accredited to ISO 15189.^2^ Kenya was well funded and equipped with a system for implementing, managing, and monitoring laboratory activities, and by 2017 had implemented SLIPTA in more than 140 laboratories. In accordance with the WHO Guide for SLIPTA implementation,^7^ the Kenya Ministry of Health SLIPTA focal office prioritised laboratories, coordinated and guided this process. Despite measurable improvements recorded by the laboratories, less than 10% had achieved ISO 15189 accreditation, with most stagnating at 2–3 SLIPTA stars.^8^

Audu et al.^9^ describe experiences in Nigeria where laboratories implementing SLMTA with expert on-site mentorship improved faster and steadier, achieving a score of 5 stars. Hospital management buy-in and strong laboratory staff camaraderie were found to be essential for the positive changes observed during SLMTA implementation in Botswana.^10^ Further, a study conducted in Ethiopia on perceptions and attitudes toward SLMTA among laboratory and hospital professionals recommended additional training and staff advocacy workshops for medical directors and hospitals’ upper management in a bid to address concerns about the duration of the process and resource allocation.^11^ A qualitative study carried out in Rwanda to better understand the reasons and context underlying decrease in laboratory performance during SLMTA implementation identified various factors, notably lack of coordination, ownership by the laboratory workforce and insufficient stakeholders’ communication, as contributors to low performance.^12^

Therefore, to accelerate the pace of medical laboratory accreditation in Kenya, the United States Centers for Disease Control and Prevention in collaboration with the Ministry of Health and other stakeholders embarked on a rapid results initiative (RRI) to re-energise the Kenya’s laboratory quality improvement process through SLIPTA. Historically, Kenya has used a 100-day RRI aggressive programming approach to attain accelerated achievement of targets that had stagnated or made slow progress. The RRI approach was applied in the HIV programme to improve uptake of the prevention of mother-to-child transmission programme and male involvement^13,14^ and uptake of voluntary medical male circumcision.^15^ This approach also has been implemented in other sectors of the Kenyan government.^16^ This project sought to fast-track through an aggressive RRI, the achievement of improved laboratory QMS and increase ISO 15189 accreditation among medical laboratories whose quality management improvements had stagnated based on SLIPTA assessment after more than two years of SLMTA implementation. In this article, we describe Kenya’s experience with accelerating medical laboratories on SLMTA towards attaining ISO 15189 accreditation.

## Methods

### Ethical considerations

We conducted this RRI project as part of ongoing PEPFAR-supported initiatives in Kenya to improve laboratory testing quality to support access to quality laboratory services for optimal care and treatment of persons with HIV and tuberculosis. Data collection and analysis were part of routine laboratory strengthening programmes approved by the Kenya Ministry of Health. The study was approved by the Kenyatta National Hospital – University of Nairobi Ethical Review Committee (KNH-UON#: P723/10/2018). This project was further reviewed by the United States CDC, Center for Global Health, Associate Director for Science in accordance with CDC human research protection procedures and was determined to be non-research (CGH-HSR#: 2018-355).

### Rapid results initiative taskforce

In March 2017, the Ministry of Health in Kenya set up a RRI taskforce to review and advise on Kenya’s progress in implementing quality systems improvement through SLIPTA, among laboratories enrolled in the SLMTA programme since 2010. The RRI taskforce included representatives from the United States President’s Emergency Plan for AIDS Relief (PEPFAR), Ministry of Health and the SLIPTA focal office, Centers for Disease Control and Prevention and PEPFAR implementing partners, with extensive knowledge and experience in the stepwise laboratory QMS strengthening initiatives in Kenya. As part of the deliverables, the RRI taskforce reviewed all 140 SLMTA-implementing laboratories and selected 39 that had stagnated in the SLMTA programme for more than 2 years without achieving accreditation to ISO 15189 standard. To accelerate progress towards accreditation of these laboratories that had gone through the full cycle of standard SLMTA without success, the taskforce provided the following as part of the RRI process: high-level advocacy, upper management and facility stakeholder engagements, RRI on-site support and mentorship, training, laboratory audits and RRI technical assistance visits.

### High-level advocacy, upper management and facility stakeholder engagements

The RRI taskforce convened two-tier engagement meetings with stakeholders and health facility personnel in March 2017. The first tier of meetings were at national and county-level and entailed advocacy engagement workshops that drew attendees from national and county laboratory leadership: Ministry of Health Division of Laboratory Services, Kenya National Accreditation Service, County Directors of Health, County Medical Laboratory Coordinators and PEPFAR laboratory programme implementing partners. This was done to gain consensus and commitment for support from the stakeholders as a top-down approach. The second tier of meetings were held in several regions bringing together top county-level laboratory leadership, upper health facility management, and laboratory leadership from the targeted laboratories. Typically, attendees of the regional meetings included County Directors of Health, hospitals’ medical superintendents, County Medical Laboratory Coordinators, facility laboratory managers, laboratory equipment maintenance staff, human resource officers, procurement officers and laboratory quality assurance officers. At each regional meeting, the RRI taskforce discussed the key steps for the RRI process and need to provide additional resources to address gaps identified. Each regional meeting also served as an official kick-off for the RRI process at the selected facilities.

### Rapid results initiative on-site support and mentorship

For all the laboratories on RRI, mentorship was targeted based on SLIPTA baseline audit findings, gaps were rapidly addressed following the SLMTA process but in shortened timelines, because the laboratories had already gone through the standard SLMTA training. The aggressive mentorship entailed a review and in some cases revision of the basic quality documents such as the quality manual, standard operating procedures, and test methods. These documents were then used to guide the rest of the RRI process to raise the laboratory performance on the 12 quality system essentials (QSEs) which include: Documents & Records (QSE 1), Management Reviews (QSE 2), Organization & Personnel (QSE 3), Client Management & Customer Service (QSE 4), Equipment (QSE 5), Evaluation and Audits (QSE 6), Purchasing & Inventory (QSE 7), Process Control (QSE 8), Information Management (QSE 9), Identification of Non Conformities, Corrective and Preventive Actions (QSE 10), Occurrence/Incident Management & Process Improvement (QSE 11) and Facilities and Biosafety (QSE 12).

### Trainings

Targeted SLMTA-driven QMS improvement trainings were conducted at either central or facility-level based on cross-cutting needs among laboratories on the RRI (Table 1). Trainings conducted at each site were recommended by laboratory quality systems experts informed by the gaps identified during the baseline assessments and internal audits. The trainings were facilitated by SLMTA-trained trainers and experienced laboratory mentors drawn from the Ministry of Health and implementing partners. The trainings entailed a mix of didactic instructions and hands-on demonstrations in the laboratories.

**TABLE 1:** Trainings and QSE-based mentorship interventions used to fast-track implementation of quality management system and monitoring by SLIPTA in 39 laboratories in Kenya, 2017–2019.

Setting	QSE areas	Activity
**Central level**	ISO 15189 management requirements	Interpreting management sections of the ISO 15189
	SLMTA and quality management system training for laboratory managers	Overview of SLIPTA checklist, review of emerging challenges and mitigation during lab quality management system implementation
	Management reviews	Laboratory work plan and resource allocation – synergy across management responsibilities, laboratory audits, feedback, corrective actions and quality laboratory services
		Personnel management responsibilities
	Internal audits	Refresher on conducting internal audits using SLIPTA checklist
**Facility-level**	Documents and records	Hands-on updating of laboratory standard operating procedures, policies, personnel records and aligning to ISO 15189 standard
	Organisation and personnel	Training on laboratory organisation, staffing matrix, continuous education, competency assessments, appraisals, and communication for quality services
	Management reviews	Review of management responsibilities for laboratory quality management system, on relationship between laboratory audits, customer satisfaction, quality indicators, corrective actions and feedback loops
	Process control	Practical review of quality control and external quality assessment implementation, identification of barriers, outcomes, root cause analysis and corrective actions for unacceptable external quality assessment results
	Information management	Refresher on reporting of validated laboratory results and use of verified laboratory information system to manage laboratory reports
		Reporting summary data to national systems and use of data for decision-making
	Identification of nonconformities and corrective actions	Mentorship on conducting internal laboratory audits, root cause analysis and corrective actions
	Equipment management	Mentorship on maintenance of equipment files, verification and maintenance

SLIPTA, Stepwise Laboratory Improvement Process Towards Accreditation; QSE, quality system essential.

### Laboratory audits

Two external audits, baseline in April 2017 and exit in July 2017, were conducted at each laboratory using the e-SLIPTA checklist (adapted from the official WHO AFRO SLIPTA checklist^6^), which was loaded on tablets with Open Data Kit (Get ODK Inc., San Diego, California, United States). For this initiative, utility of e-SLIPTA checklist was to enhance rapid transmission and analysis of audit data. The short turnaround time of two days for audit report summaries achieved using the e-SLIPTA checklist expeditiously completed the communication loop quickly providing information on identified gaps for targeted SLIPTA mentorship towards their fast-tracked resolution. Each audit lasted one day and was conducted by experienced laboratory auditors who had been trained on using the e-SLIPTA checklist. The laboratory audit team reviewed quality and technical records, observed laboratory practices and discussed QMS practices with the laboratory staff. The auditors documented areas of non-conformity and, per the SLIPTA guidance, provided on-site technical assistance and recommendations for mitigating identified gaps. Results of the laboratory audits were recorded for each of the 12 sections of the checklist, which covers the 12 QSEs (CLSI GP 26-A3^17^). All audits and laboratory rating classifications were performed as per the WHO AFRO SLIPTA checklist.^6^ The checklist has 334 questions for a total of 275 possible points. At the end of each audit, the scores were analysed and presented to each individual laboratory on follow-up technical visits. The laboratories were tasked to perform internal audit at RRI mid-term and as needed following resolution of most nonconformities identified at previous audit. The mid-term internal audit was used by the RRI taskforce to conduct additional facility technical visits for both the hospital management and the laboratory. Laboratories that scored ≥ 2 stars at exit audit were recommended for the 6-month post-RRI mentorship period in Phase 3. Laboratories scoring SLIPTA ≥ 3 stars at any time during the RRI process were recommended to initiate the Kenya National Accreditation Service ISO 15189 accreditation application process. Kenya National Accreditation Service accreditation was achieved in Phase 3 (the post-RRI period) following ISO 15189 accreditation assessments and closure of assessment gaps.

### Rapid results initiative technical assistance visits

Members of the RRI taskforce conducted technical site visits to the target laboratories following baseline audits and provided on-site technical assistance and recommendations for closing identified gaps. First came meetings with laboratory staff, mentors and PEPFAR implementing partners providing moral support and technical details for resolving highlighted SLIPTA audit gaps. Second were the technical site visit meetings with top management which utilised the audit report scores to advocate for support to the laboratory from the health facility and county administration and management offices. Advocacy to the upper management was focused on addressing deficiencies related to management review meetings, infrastructure, and resource mobilisation aimed at meeting quality-related gaps in the laboratories. Additionally, the PEPFAR partners working at those facilities provided one-off support for nonconformities that required additional financing beyond what the facility and county management teams were able to resolve.

### Period of implementation

The RRI process was implemented in three phases:

- Phase 1: This was a pre-RRI planning and activation period of 2 months (April 2017 – June 2017) that entailed high-level advocacy, and national and county stakeholder engagement, national and county-level trainings, external and internal audit refresher trainings for SLMTA auditors, and deploying the e-SLIPTA checklist.
- Phase 2: This was the aggressive, facility-level RRI implementation period of 100 days (July 2017 – October 2017) that entailed facility-level advocacy and hospital management engagement, laboratory QMS trainings, targeted laboratory mentorships and laboratory audits using the e-SLIPTA checklist. Baseline and exit external audits were conducted at the beginning and at the end of this phase. The RRI process implementation support, aimed at closing gaps identified by SLIPTA audits, was standardised across all 39 laboratories.
- Phase 3: This was the post-RRI period of six months (October 2017 – March 2018) that entailed follow-up ongoing mentorship to close gaps and nonconformities identified by RRI exit audits and 12 months (April 2018 – March 2019) of monitoring achievement of accreditation to ISO 15189 as the outcome of interest for eligible facilities.

### Data analysis

To describe QMS performance of laboratories participating in the RRI, proportions of laboratories at different star ratings before and after RRI interventions were computed based on the two external RRI audits conducted at baseline and at exit. Average QSE point audits scores and respective percentage scores were calculated and presented as means with standard deviation (s.d.) and as percentages with their 95% confidence intervals (CI). To describe improvements in the laboratory QMS, QSE-specific mean point scores between baseline and exit audits were compared using a paired t-test to assess changes in performance. Radar charts (spider graphs) of average baseline and exit QSE percentage scores were plotted to demonstrate relative improvement in QSE performance following RRI interventions. To tease out some of the challenging QSEs that did not make significant improvements despite the RRI process, QSE performance was compared between laboratories that moved upwards by 2 or more stars from baseline audit star rating to exit audit star rating (i.e. made a stepwise move upwards of ≥ 2 star steps; e.g. from 0 stars at baseline to 2 stars at exit or 1 star at baseline to 3 stars at exit and so on) and those that did not (moved upwards < 2 star steps). Average percentage score changes or mean point score increases between baseline and exit audits with *p*-value < 0.05 were considered statistically significant. Analysis was performed using Stata statistical software, version 12.0 (Stata Corp., College Station, Texas, United States).

## Results

### Overall laboratory Stepwise Laboratory Improvement Process Towards Accreditation star rating performance

**FIGURE 1:**
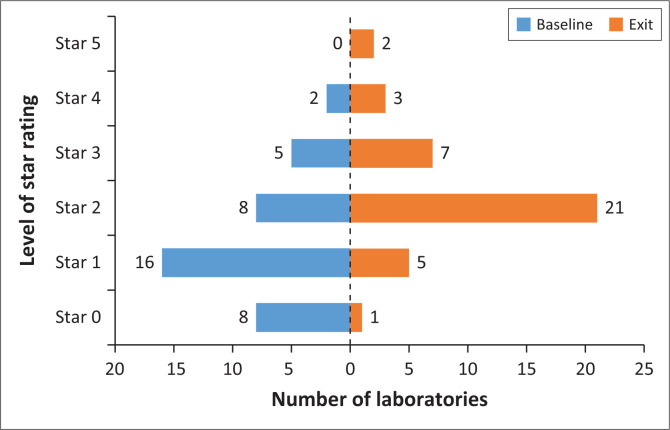
Laboratory star rating before and after rapid results initiative implementation of Strengthening Laboratory Management Toward Accreditation in 39 laboratories in Kenya, 2017–2019.

All of the 39 laboratories targeted for RRI process were in urban settings. Of these, 32 (82%) were Ministry of Health-owned, while seven (18%) were affiliated to faith-based organisations (FBOs). All 39 laboratories enrolled in the RRI completed the intensive mentorship sessions including scheduled baseline and exit external audits (Table 1). At baseline audit, most laboratories (24/39, 62%) were below 2 stars, eight (21%) were rated at 2 stars, and only seven (18%) were at ≥ 3 stars. At the exit audit, only six (15%) laboratories scored below 2 stars, 21 (54%) were at 2 stars, and 12 (31%) achieved ≥ 3 stars (Figure 1). Therefore, the number of laboratories that scored ≥ 2 stars rating increased significantly from 15 (38%) at baseline to 33 (85%) at RRI exit (*p* < 0.001). There was no significant difference in performance between Ministry of Health-owned and FBO-affiliated laboratories, of which 75% and 71% achieved ≥ 2 stars rating. Overall, 34 (87%) laboratories achieved the desired goal of ISO 15189 accreditation in less than two years of the RRI process. This resulted in a 330% increase in the number of ISO-accredited laboratories (from 15 laboratories in June 2017 to 49 laboratories in June 2019) through PEPFAR funding in Kenya.

### Mean changes in quality system essentials scores during RRI

The performance of the 12 QSEs before and after the RRI is displayed in Table 2. Of the 35 possible points assigned to Equipment Management (QSE 5) on the WHO SLIPTA checklist, the mean (s.d.) baseline score for the 39 RRI laboratories was 21.97 (4.31) and increased to 26.28 (4.07) at exit. This QSE experienced the most significant mean increase of 5.31 ± 1.89 (*p* < 0.0001). Facilities and Biosafety (QSE 12) with a mean increase of 4.05 ± 1.78 (*p* < 0.0001) and Management Reviews (QSE 2) with a mean increase of 3.18 ± 1.77 (*p* < 0.001) were the second and third most impacted QSEs by the RRI interventions (Table 2). Client Management & Customer Service (QSE 4) (*p* = 0.0224) and Occurrence/Incident Management & Process Improvement (QSE 11) (*p* = 0.0277) also had significant mean point increases. Although Evaluation and Audits (QSE 6) had the second-highest percent increase in performance, the mean point increase from baseline to exit of 1.51 ± 2.24 for this QSE was not statistically significant (*p* = 0.1581).

**TABLE 2:** Quality system essentials score changes following RRI for implementation of QMS through SLIPTA in *39* laboratories in Kenya, 2017–2019.

Quality system essential	Total points	Baseline score		Exit score		Mean change ± 95% CI	*p*
		Mean	s.d.	Mean	s.d.		
QSE 1: Documents & Records	28	17.54	5.57	19.15	4.22	1.62 ± 2.23	0.1294
QSE 2: Management Reviews	14	4.03	3.83	7.21	4.02	3.18 ± 1.77	0.0017
QSE 3: Organisation & Personnel	22	15.36	3.79	16.72	3.85	1.359 ± 1.72	0.1553
QSE 4: Client Management & Customer Service	10	7.13	2.03	8.03	1.39	0.90 ± 0.79	0.0224
QSE 5: Equipment Management	35	20.97	4.31	26.28	4.07	5.31 ± 1.89	< 0.0001
QSE 6: Evaluation and Audits	15	6.15	5.06	7.67	4.85	1.51 ± 2.24	0.1581
QSE 7: Purchasing & Inventory	24	17.62	4.73	19.08	3.99	1.46 ± 1.97	0.0900
QSE 8: Process Control	32	19.59	6.81	21.64	3.80	2.051 ± 2.50	0.1012
QSE 9: Information Management	21	11.31	3.48	11.36	1.99	0.05 ± 1.28	0.9288
QSE 10: Identification of Nonconformities and Corrective and Preventive Actions	19	8.69	4.73	8.23	4.59	0.46 ± 2.10	0.5788
QSE 11: Occurrence/Incident Management & Process Improvement	12	7.33	2.79	8.92	2.87	1.59 ± 1.28	0.0277
QSE 12: Facilities and Biosafety	43	33.46	4.62	37.51	3.11	4.05 ± 1.78	< 0.0001

QSE, quality system essential; s.d., standard deviation; CI, confidence interval.

### Quality system essential performance-related laboratory star improvement

**FIGURE 2:**
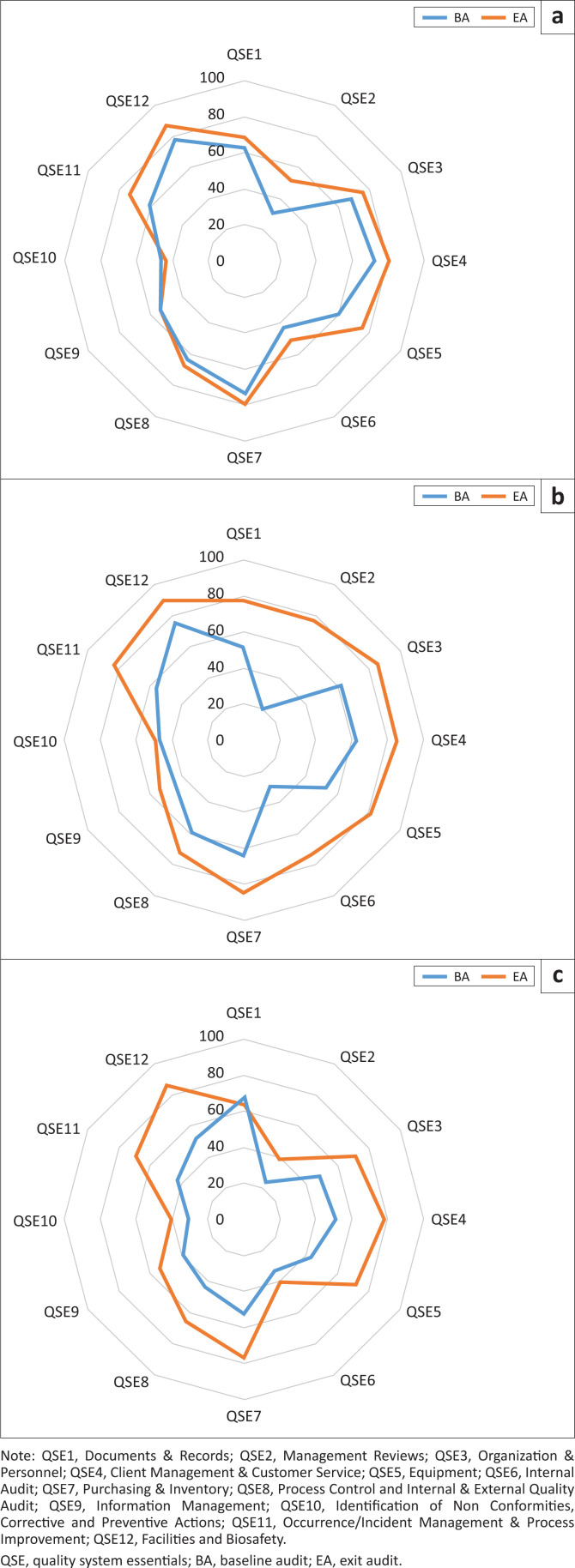
Average percent score for quality system essentials performance before and after 100-day rapid results initiative for laboratories to fast-track Stepwise Laboratory Improvement Towards Accreditation in 39 laboratories in Kenya, 2017–2019. (a) All laboratories (*n* = 39); (b) laboratories with ≥ 2-step star increase (*n* = 13); (c) laboratories with < 2-step star increase (*n* = 26).

Of the 12 QSEs, Management Reviews (QSE 2), Evaluation and Audits (QSE 6), Information Management (QSE 9) and, Identification of Nonconformities, Corrective and Preventive Actions (QSE 10) were overall the most challenging to improve toward the maximum achievable respective scores, barely crossing the 50.0% mark after RRI interventions. The baseline average percentage scores were 30.0% for QSE 2, 43.0% for QSE 6, 54.0% for QSE 9, and 47.0% for QSE 10, compared to 52.0%, 51.0%, 54.0%, and 43.0% at RRI exit (Figure 2a). To understand QSEs that were most problematic despite the RRI efforts, we assessed QSE level performance for 13 (33.0%) of the 39 laboratories that improved by 2-star steps compared to the other 26 (67.0%) laboratories that did not (i.e. moved upwards < 2-star steps) as shown in Figure 2b and 2c. In the first stratum of these 13 laboratories that attained ≥ 2-star step increases, the average percentage scores for all QSEs increased substantially following RRI interventions, except for QSE 10, which only had minimal improvement from 47.0% at baseline to 49.0% at exit (Figure 2b). In these 13 laboratories, QSE 2 and QSE 6 were the most improved with an increase in performance from 20.3% (95% CI: 5.2% – 50.1%) to 76.9% (95% CI: 47.3% – 93.5%) and 28.7% (95.0% CI: 10.1% – 57.1%) to 73.3% (95.0% CI: 44.8% – 91.1%), despite being among the most challenging QSEs to improve when all 39 laboratories were pooled together. QSE 9 and QSE 10 remained the most difficult to improve across the 13 laboratories included in this stratum (Figure 2b), barely crossing the 50.0% mark. Conversely, among the 26 laboratories in the second stratum, QSE 2, QSE 6, and QSE 10 had the least improvement in performance, barely crossing the 40.0% mark after RRI interventions. Therefore, these three QSEs in addition to QSE 9 remained the most challenging among the 26 laboratories that increased < 2-star step despite RRI interventions (Figure 2c). Although overall star rating performance for the second stratum of 26 laboratories was not substantial, several QSEs improved due to the RRI interventions (Figure 2c). QSE 4, QSE 5, QSE 11, and QSE 12 had the highest increase in average percentage scores, ranging from 27.0% to 33.0%.

## Discussion

Our findings confirm that aggressive high-level advocacy and management buy-in, targeted mentorship and site-level support implemented in 100 days followed by an additional 6-month ongoing mentorship resulted in a 330% increase in number of ISO 15189 accredited medical laboratories in Kenya. To our knowledge, our report is the first to describe expedited improvement of laboratory quality systems for laboratories on the SLMTA programme through RRI.

Several factors can be cited for the rapid improvement of laboratories that implemented RRI. The QMS intervention trainings that were conducted at facility and centralised levels renewed the stakeholders’ interest in the ISO 15189 accreditation and motivated the laboratory staff to ‘sprint toward accreditation’. The RRI was implemented by a coalition of laboratory experts in quality systems implementation that ensured inter-mentor strengths across the 12 QSEs, while maintaining accountability and focus on targeted interventions. Further, by leveraging on the strengths of each mentor, this arrangement provided the much-needed capacity to rapidly execute the RRI process in 39 laboratories spread across the country using a relatively small team.

During the implementation process, mentors provided laboratory performance and non-conformance data to upper management for action, ensuring continuous communication with upper management. This in turn provided commitment and resources needed for laboratory staff to address emerging challenges within a short time. Our experience accords with the available reports on the critical role of laboratory mentorship and advocacy in helping laboratories achieve accreditation goals during the SLIPTA process.^18,19^ An additional success factor for this initiative was the adoption of Kenya’s version of the e-SLIPTA checklist to audit laboratories. The e-SLIPTA checklist provided an interactive dashboard for identifying gaps and improving laboratory performance in real time. This simplified the feedback of audit findings to upper hospital management for prompt action.^20^

We found that of the 12 QSEs monitored during the RRI initiative, improvement in QSE 2 (Management Reviews), QSE 6 (Evaluation and Audits), and QSE 10 (Identification of Nonconformities, Corrective and Preventive Actions) were critical for any laboratory to demonstrate a 2-star improvement after 100 days. This observation is consistent with previous findings that identified upper management support for laboratory improvement, adequate audit skills and planning as critical factors for high audit scores and corrective actions necessary for laboratories to achieve accreditation.^21,22,23^ The RRI taskforce addressed these obstacles before and during RRI implementation. Initial trainings and mentorships served to plan and build staff audit skills while sharing of audits findings through e-SLIPTA ensured timely implementation of corrective and preventive actions.

A significant number (67%) of the laboratories that implemented RRI recorded a less than 2-step star improvement after the RRI, with some stagnating at the 1-star level or below. Poor performance and stagnation in some laboratories were linked to three QSEs: Management Reviews (QSE 2), Evaluation and Audits (QSE 6), and Identification of Nonconformities, Corrective and Preventive Actions (QSE 11). These results were unexpected, because the RRI interventions had been designed primarily to address these QSEs based on previous observations of most laboratories initially progressing rapidly toward meeting the SLMTA goals but stagnating between 2 and 3 stars, while others regress to 1 or 0 star (Unpublished work; Makokha et al.). In addition, most of the staff in the RRI-targeted laboratories were experienced in implementing laboratory QMS and had received refresher training in corrective action, occurrence management, and internal audits as part of this project. A low score in Information Management (QSE 9) among the laboratories that recorded less than 2-star improvements after the RRI process was unexpected because all the laboratories implementing RRI had functional laboratory information systems. However, QSE 9 was flagged as a stagnating factor, because the 100 days within which the RRI was implemented were inadequate to address it.

### Limitations

This project had several limitations. Although implementation of the RRI process achieved tangible improvements in QMS and overall quality within a short time, these results were based on a small cohort of laboratories that had previously received SLMTA trainings and implemented several QSE improvements. The RRI was implemented in a group of laboratories with cumulative experience of both success and stagnation in improving quality through SLMTA. Another limitation is that we did not analyse the effect of laboratory staff levels and their experience with the RRI process and outcomes. Hence, these results cannot be easily generalised, because previous experience may have facilitated rapid improvements in some of the laboratories.

### Conclusion

With the right advocacy strategy, mentorship teamwork, leadership buy-in, and effective communication, implementation of laboratory QMS improvement through SLMTA and assessed by SLIPTA could benefit from RRI catalysing accreditation preparedness for medical laboratories. Engaging facility management and county leadership throughout the process of laboratory accreditation is key to closing nonconformities identified during mentorship. The RRI led to several carry-over effects well beyond the 100 days and increased the number of laboratories achieving accreditation to the ISO 15189 standard in Kenya.
